# Infection Characteristics of Rice Stripe Mosaic Virus in the Body of the Vector Leafhoppers

**DOI:** 10.3389/fmicb.2018.03258

**Published:** 2019-01-08

**Authors:** Ping Zhao, Xiang Sun, Pan Li, Jiatao Sun, Yue Yue, Jing Wei, Taiyun Wei, Dongsheng Jia

**Affiliations:** Fujian Province Key Laboratory of Plant Virology, Institute of Plant Virology, Fujian Agriculture and Forestry University, Fuzhou, China

**Keywords:** cytorhabdovirus, rice stripe mosaic virus, leafhopper, infection route, vector

## Abstract

Rice stripe mosaic virus (RSMV), a novel species of *Cytorhabdovirus*, is transmitted by the leafhopper *Recilia dorsalis* in a persistent-propagative manner. In this study, we firstly confirmed that N protein of RSMV is a component of viroplasm and virion in vector culture cells of *R. dorsalis*. Confocal microscopy revealed that RSMV initially accumulated in epithelial cells of the filter chamber of *R. dorsalis*, from where it proceeded to the visceral muscles surrounding the filter chamber. Subsequently, RSMV spread quickly throughout the suspensory ligament to the salivary glands. Meanwhile, RSMV spread from the filter chamber to midgut, hindgut, esophagus, hemolymph, and central nervous system. We further observed that RSMV particles displayed as non-enveloped form when propagating in cytoplasm of different tissues, and became enveloped when spread within insect body by electron microscopy. Additionally, we found that the leafhopper *Nephotettix virescens* was also able to acquire and transmit RSMV. These results clarified the infection characteristics of RSMV in its leafhopper vectors, which will help guide the formulation of RSMV prevention and control strategies.

## Introduction

Many plant viruses are transmitted by sap-sucking insects including thrips, aphids, planthoppers, leafhoppers, and whiteflies in a persistent manner (Hogenhout et al., 2008). After ingestion, the persistent plant viruses enter the gut epithelium from the gut lumen and release into the hemolymph or other tissues of insect vectors, finally invade the salivary glands, from where they are introduced back into the plant host during insect feeding (Hogenhout et al., 2008). During the process, they must overcome multiple barriers, including midgut infection/escape barrier and salivary gland infection/escape barrier in vector insect (Hogenhout et al., 2008). Midgut represents the first bottleneck for viral infection, and salivary gland is the key barrier for viral transmission (Chen and Wei, 2016; Jia et al., 2018). However, the mechanisms of how persistent plant viruses overcome the midgut and salivary gland barriers remain poorly understood. Recently, it has been well documented that the virus-associated filaments or tubules have been used by persistently transmitted plant viruses, such as plant reoviruses (family Reoviridae) to overcome the midgut, salivary gland, and ovary barriers of insect vectors (Chen et al., 2012; Jia et al., 2014; Wu et al., 2014; Liao et al., 2017; Mao et al., 2017). Meanwhile, the hemolymph, nervous system, tracheal, muscle tissues, or cellular structures providing the direct contact between the midgut and salivary gland have been used by persistently transmitted plant viruses as the routes for spread from midgut to salivary gland of insect vectors (Whitfield et al., 2005; Ammar and Hogenhout, 2008; Ammar et al., 2009; Montero-Astúa et al., 2016). Acquiring a better understanding of this infection route of plant viruses and the barriers that they encounter in their insect vectors should lead to the development of better strategies to block virus transmission.

Plant rhabdoviruses are usually transmitted by hemipteran insects in a persistent-propagative manner (Ammar et al., 2009). Replication and transcription/translation of rhabdoviruses is consistent across different viruses in the family (Walker et al., 2011). The N proteins function in the encapsidation of the viral genomic RNA and are part of viroplasms and polymerase complexes (Luo et al., 2007). After entry into the host cell and release of the nucleocapsid core (NC: RNA-N-P-L), the rhabdovirus polymerase complex initiates transcription. Replication is thought to be initiated after the N protein accumulates to a sufficient concentration to mediate a switch from transcription to replication (Dietzgen, 2012). Rice stripe mosaic virus (RSMV), a new cytorhabdovirus (family Rhabdoviridae), is transmitted by the leafhopper *Recilia dorsalis* in a persistent-propagative manner (Yang et al., 2017a). The bacilliform, enveloped viral particles of RSMV are 300 to 375 nm long and 45 to 55 nm wide, and contain a single negative-sense RNA that encodes seven canonical proteins in the following conserved order: nucleocapsid protein (N), phosphoprotein (P), non-structural protein P3, matrix protein (M), glycoprotein (G), non-structural protein P6, and large polymerase protein (L) (Yang et al., 2017a). RSMV was first discovered in Guangdong, China in 2015, and has spread to the neighboring provinces in 2017, which has caused serious yield losses in rice production (Yang et al., 2017a). Although the virus particle morphology, molecular biology, and transmission biology of RSMV have been well studied (Yang et al., 2017b), the infection mechanism of RSMV in the leafhopper vector remains a mystery. Hence, in this study, immunofluorescence and electron microscopy assays were used to investigate the infection characteristics of RSMV in its leafhopper vectors and the ability of the leafhopper *Nephotettix virescens* and *Nephotettix cincticeps* to transmit RSMV.

## Materials and Methods

### Virus, Cell, and Insects

RSMV samples, collected from rice fields from Luoding, Guangdong, China, were maintained on rice plants via transmission by *R. dorsalis*. Continuous cultures of the vector cell in monolayer (VCM) were developed from *Recilia dorsalis* cells and maintained on the growth medium as described previously (Chen et al., 2013). RSMV samples, collected from rice fields from Luoding, Guangdong, China, were maintained on rice plants via transmission by *R. dorsalis*. The particles of RSMV were purified from RSMV-infected rice plants as described previously (Yang et al., 2017a). Briefly, 500 grams of RSMV-infected rice leaves were ground in extraction buffer (100 mM Tris-HCl, pH 8.0). After filtered and centrifuged, the supernatant was ultra centrifuged (45,000 rpm) for 30 min at 4°C.The pellets were resuspended in 10 mL extraction buffer and the suspension was centrifuged by sucrose density gradient centrifugation. The light band was collected and ultra centrifuged, and the pellet was resuspended in 2 mL resuspension buffer (50 mM Tris-HCl, pH 8.0; Yang et al., 2017a). The purified RSMV from infected rice were used to infect the VCMs as described previously (Chen et al., 2013). Non-viruliferous populations of *R. dorsalis, N. virescens*, and *N. cincticeps* were collected from healthy field plants in Xingning, Guangdong, China. They were propagated on healthy rice plants grown in insect-proof greenhouse at 25 ± 1°C, under conditions of 75 ± 5% relative humidity and a photoperiod of 16 h of light and 8 h of darkness, as described previously (Chen et al., 2016).

### Antibody Preparation

The mouse polyclonal antiserum against N protein of RSMV was prepared, as described previously (Jia et al., 2012b). Briefly, the complete open read frame of N gene from RSMV was amplified by RT-PCR and engineered into Gateway vector pDEST17. The resulting plasmid pDEST17-N was transformed into *Escherichia coli* Rosetta strain and expressed by adding isopropyl-β-D-thiogalactopyranoside (IPTG). The N protein was purified with Ni-NTA resin and immunized into mouse as described previously (Jia et al., 2012b). IgG was isolated from specific polyclonal antiserum using a protein A-Sepharose affinity column and conjugated directly to rhodamine according to the manufacturer's instructions (Invitrogen). In order to detect the specificity of N antibody of RSMV, the total plant protein extracts were prepared and detected by Western blot as described previously (Jia et al., 2012b).

### Immunofluorescence Staining of the Organs of *R. dorsalis* After Acquisition of Virus

To determine the infection route of RSMV by the leafhopper *R. dorsalis*, the 2rd instar nymphs from non-viruliferous populations of leafhoppers (*n* = 50, three biological repetitions) were fed for 2 days on RSMV infected rice plants, then transferred to healthy rice seedlings. At 2, 4, 6, 8, and 12 days post-first access to diseased plants (padp), the digestive systems, compound ganglionic mass and reproductive system (50 insects/time point) were dissected, fixed in 4% paraformaldehyde, permeabilized in 2% Triton X-100, stained with N-rhodamine, phalloidin-Alexa Fluor 488 carboxylic acid, and then examined with a Leica TCS SP5 inverted confocal microscope, as described previously (Chen et al., 2011). In addition, we collected the hemolymph from 30 viruliferous *R. dorsalis* at different day padp as described previously (Jia et al., 2012a). The hemolymphs were fixed and stained with N–FITC (green) and DAPI (blue), and then examined with confocal microscope as described above.

### Electron Microscopy

The RSMV infected VCMs on coverslips were fixed, dehydrated, embedded, and thin sections were cut as described previously (Wei et al., 2009). Cell sections were then incubated with antibodies against RSMV-N and immunogold labeled with goat antibodies against rabbit IgG that had been conjugated to 15 nm gold particles (Sigma). As controls, mock-infected VCMs were treated in the same way with virion resuspension buffer. Meanwhile, the digestive systems, central nervous, and salivary glands of RSMV infected *R. dorsalis* were fixed, dehydrated and embedded, and thin sections were cut as described above. Thin sections were examined with a Hitachi H-7650 transmission electron microscope. As controls, the tissues dissected from *R. dorsalis* that had fed on healthy rice plants were treated in the same way.

### RSMV Acquisition and Transmission Ability of *N. cincticeps* and *N. virescens*

To determine whether RSMV can be acquire and transmit by *N. cincticeps* and *N. virescens*, the method reported by Chen et al. (2016) was followed. Briefly, the 2rd instar *N. cincticeps* and *N. virescens* nymphs (*n* = 50, three biological repetitions) were fed on RSMV-infected rice plants for 2 d and then placed on healthy rice seedlings for 14 days. Then the digestive systems of insects were analyzed by immunofluorescence staining detection with N-FITC as previously. Meanwhile, individual leafhopper adults were fed on healthy rice seedlings in individual tubes. The tested plants were replaced by healthy ones every 2 d until the insects died. The dead insects were timely collected and tested *N* gene by the RT-PCR for RSMV infection. Briefly, total RNA (0.5 μg) from single insect was reverse-transcribed using RevertAid Reverse Transcriptase (Thermo) in a 10-μl volume and 1 μl cDNA were used for PCR detection. The specific primers for *N* gene were N-F (5′-ATGGCAACCGACAAGTCTTTTG-3′) and N-R (5′- CTTGGCTGTATCATCCTCTTCGT-3′). The PCR parameters consisted of one cycle at 95°C for 5 min, followed by 34 cycles of 95°C for 30 s, 55°C for 30 s, and 72°C for 90 s. The seedlings were grown further for observation of disease symptoms and detection by RT-PCR as described above.

## Results

### RSMV Particles Accumulated Around the Viroplasm in RSMV-Infected Insect VCMs

**Figure 1:**
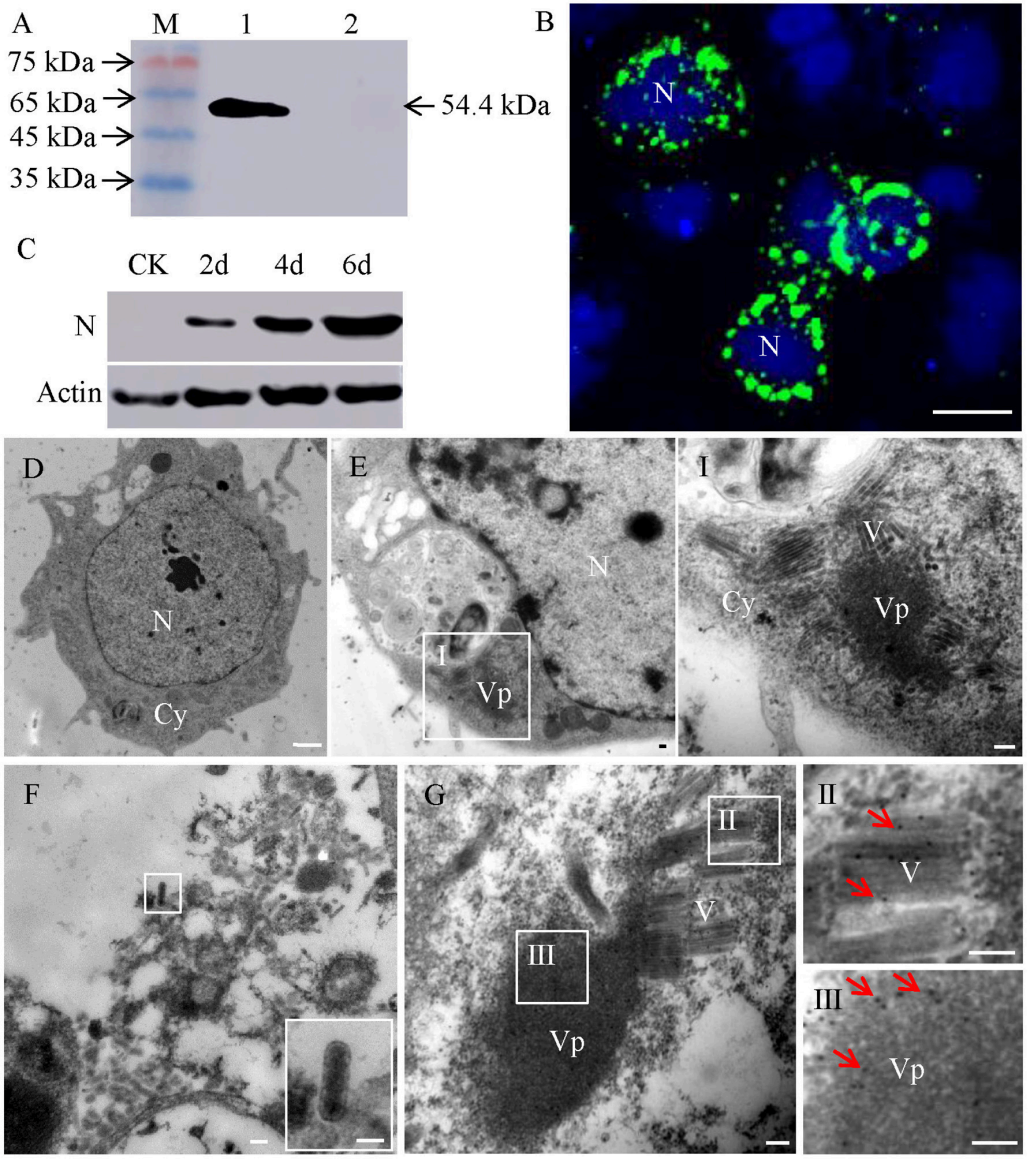
Subcellular localization of protein N of RSMV in virus-infected VCMs. **(A)** Western blot analyses of N protein. Lane M, protein marker; lane 1, protein extracts from RSMV-infected leafhopper; lane 2, protein extracts from healthy leafhopper. **(B)** Immunofluorescence staining of N-FITC (green) and DAPI (4,6-diamidino-2-phenylindole) (blue) revealed punctate inclusions in cytoplasm of RSMV-infected VCMs at 48 h p.i. Bar, 100 μm. **(C)** Accumulation of N protein in 2, 4 and 6 days p.i. CK, mock-infected VCMs. **(D)** Electron micrograph of healthy cell of *R. dorsalis*. Bar, 2 μm. **(E)** Electron micrographs of RSMV-infected cell of R. dorsalis. Panel I was the enlarged image of the boxed areas in panel E. Viroplasm and virus particles of RSMV were distributed in cytoplasm. **(F)** Electron micrograph of virus particles distributed outside the cell. Insets show the image in the boxed area. **(G)** RSMV-infected VCMs were immunolabeled with N-specific IgG as the primary antibody, followed by treatment with 15-nm gold particle-conjugated goat antibodies against rabbit IgG as secondary antibodies. Panels II and III were the enlarged images of the boxed areas in panel G. Arrows indicate gold particles. Bars in panels E-G, I, II, and III, 100 nm.

To study the localization of RSMV in viruliferous insects, we first developed RSMV N antibody which recognized a 54.4 kDa protein present in protein extracts from viruliferous insects rather than protein from non-viruliferous insects, confirming that RSMV N antibody specifically recognized RSMV N protein (Figure 1A). Next, RSMV infection and localization in VCMs was studied using immunofluorescence and western blot. The results showed that N protein localized to punctate inclusions in the cytoplasm of VCMs of *R. dorsalis* (Figure 1B) and the protein accumulation of N were increased gradually (Figure 1C). These results suggested that RSMV has successfully infected in VCMs of *R. dorsalis*. Meanwhile, electron microscopy observations showed that the non-enveloped bacilliform virions were assembled into blocks and localized around the electron-dense viroplasm in the cytoplasm of RSMV infected cell (Figure 1E, panel I), while none bacilliform virion or electron-dense viroplasm was found in mock-infected cell (Figure 1D). We only observed the enveloped bacilliform virions outside the VCMs (Figure 1F). In addition, RSMV N antibodies specifically reacted with the matrix of the viroplasm and the bacilliform virions (Figure 1G). These results suggested that RSMV N was not only a structural protein of RSMV particle, but also a constituent of the matrix of viroplasm in RSMV-infected VCMs.

### Infection Route of RSMV in Its Vector Insect as Revealed by Confocal Microscopy

**Figure 2:**
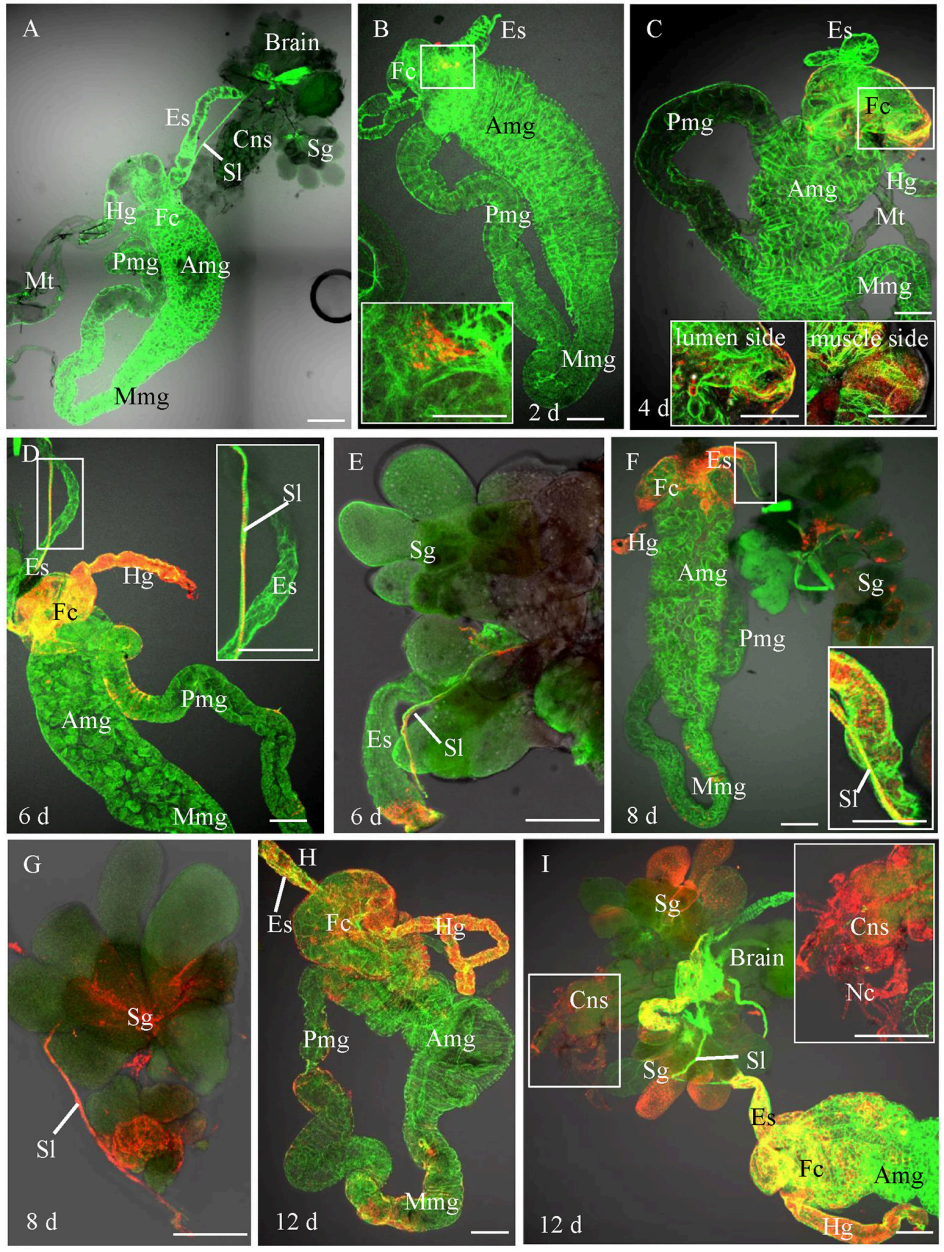
Infection route of RSMV in the insect vector. The internal organs of *R. dorsalis* were stained for viral antigens with N–rhodamine (red) and for actin with phalloidin–Alexa Fluor 488 carboxylic acid (green) and examined by confocal microscopy. **(A)** Alimentary canal of *R. dorsalis*. **(B)** At 2 day post-first access to diseased plants (padp), RSMV was detected in a small region of the filter chamber. **(C)** By 4 days padp, RSMV had spread to a wider area and around the surface of the filter chamber. **(D)** By 6 days padp, RSMV had spread from the epithelium of filter chamber to suspensory ligament, hidgut and midgut. **(E)** By 6 days padp, RSMV had spread along the suspensory ligament toward the salivary glands. **(F,G)** By 8 days padp, RSMV had infected the salivary glands. RSMV was present in the salivary glands. **(H)** By 12 days padp, RSMV spread into the visceral muscle of the midgut. **(I)** By 12 days padp, RSMV was present in the visceral muscle of the hindgut. Amg, anterior midgut; Es, esophagus; Fc, filter chamber; Hg, hindgut; Mmg, middle midgut; Mt, Malpighian tubules; Nc, nerve cord; Pmg, posterior midgut; Sl, suspensory ligament; Sg, salivary glands. Bars, 100 μm.

In order to trace the infection route of RSMV in its vector, we first observed the anatomy of organs potentially involved in virus circulation in *R. dorsalis* by confocal microscopy. *R. dorsalis'* alimentary canal was consisted of the esophagus, filter chamber, midgut, and hindgut (Figure 2A). The suspensory ligament protruded from the surface of the filter chamber, paralleled along with the esophagus, and extended to the head. The salivary gland, compound ganglionic mass, suspensory ligament, and brain connected together in the head (Figure 2A). No signal for the RSMV antigen was observed in the intestine, salivary gland, and central nerve system from the *R. dorsalis* fed on healthy rice (Figure 2A). Then, we observed the localization of RSMV-N protein in alimentary canal, salivary gland, and compound ganglionic mass of leafhoppers at different day post-first access to diseased plants (padp). At 2-day padp, RSMV N antigen was observed in a few epithelial cells of the filter chamber in about 26% of the tested insects (Figure 2B, Table 1). At 4-day padp, RSMV spread from the epithelial cells into the visceral muscle tissues of filter chamber in about 24% of the tested leafhoppers (Figure 2C, Table 1), but not spread to the hemolymph (Supplementary Figure 2B). At 6-day padp, RSMV spread to the suspensory ligament in about 32% of leafhopper samples and to the salivary glands in about 8%, suggesting that RSMV can efficiently spread from suspensory ligament to salivary glands (Figures 2D,E). Meanwhile, RSMV had spread from the filter chamber to the hidgut, midgut, and esophagus anterior in about 8, 12, and 8% of leafhoppers, respectively, but RSMV had not spread to the central nervous system (Table 1). At this time, RSMV N antigen was observed in about 20% hemolymph of the viruliferous *R. dorsalis* (Supplementary Figure 2C). At 8-day padp, RSMV had spread to the suspensory ligament and salivary glands in a higher proportion of tested leafhoppers. At this time, RSMV had spread to the central nervous system in about 20% of leafhoppers tested and 35% hemolymph of the viruliferous *R. dorsalis* (Figures 2F,G, Table 1, Supplementary Figure 2D). At 12-day padp, RSMV had spread to the entire alimentary canal, salivary glands and central nervous system in about 36% of leafhoppers (Figures 2H,I, Table 1, Supplementary Figure 2E). In the central nervous system, RSMV not only infected the compound ganglionic mass, but also distributed in the nerve cord (Figure 2I). Taken together, these results indicated that RSMV first accumulates in epithelial cells of the filter chamber, procedes to the visceral muscles surrounding the filter chamber, and then spreads throughout the suspensory ligament to salivary glands (Supplementary Figure 3). In addition, RSMV spreads simultaneously from the filter chamber to midgut, hindgut, esophagus, and hemolymph, from where RSMV spreads into the central nervous system.

**Table 1:** Occurrence of RSMV N antigens in various organs/tissues of *R. dorsalis* as detected by immunofluorescence microscopy.

**Organ/tissue examined**	**No. of insects with N antigens in different tissues (*****n*** **= 50)**				
	**2 days padp**	**4 days padp**	**6 days padp**	**8 days padp**	**12 days padp**
Filter chamber epithelium	13	18	20	19	18
Visceral muscle of Filter chamber	0	12	20	19	18
Suspensory ligament	0	0	16	19	18
Esophagus	0	0	4	12	18
Salivary gland	0	0	6	13	16
Midgut	0	0	4	10	18
Hidgut	0	0	10	17	18
Central nervous system	0	0	0	10	18

To further determine whether RSMV could be transovarially transmitted to insect offspring, immunofluorescence microscopy was used to trace RSMV N antigens in the reproductive system of leafhoppers at 20-day padp. RSMV-N antigens were observed on the surface cells of reproductive system: in the ovarioles and oviduct of females or the testis, accessory gland, and seminal vesicle of males, but did not appear in cytoplasm of the oocyte or cavity of the testis and seminal vesicle (Supplementary Figure 1). Furthermore, we traced RSMV-N antigens in the offspring of viruliferous leafhoppers with immunofluorescence staining and RT-PCR. However, no signal for the RSMV antigen was observed in the intestine, salivary gland, and central nerve system from the offspring of *R. dorsalis*. Meanwhile, RSMV could not be detected in the offspring from RSMV positive females *R. dorsalis* by RT-PCR (Supplementary Table 1). Thus, our findings suggested that the inability of *R. dorsalis* to transmit RSMV to offspring (Yang et al., 2017b) could be due to the ovary barrier that restricted viral dissemination into the oocyte.

### Infection Characteristics of RSMV in Its Vector Insect as Revealed by Transmission Electron Micrographs

**Figure 3:**
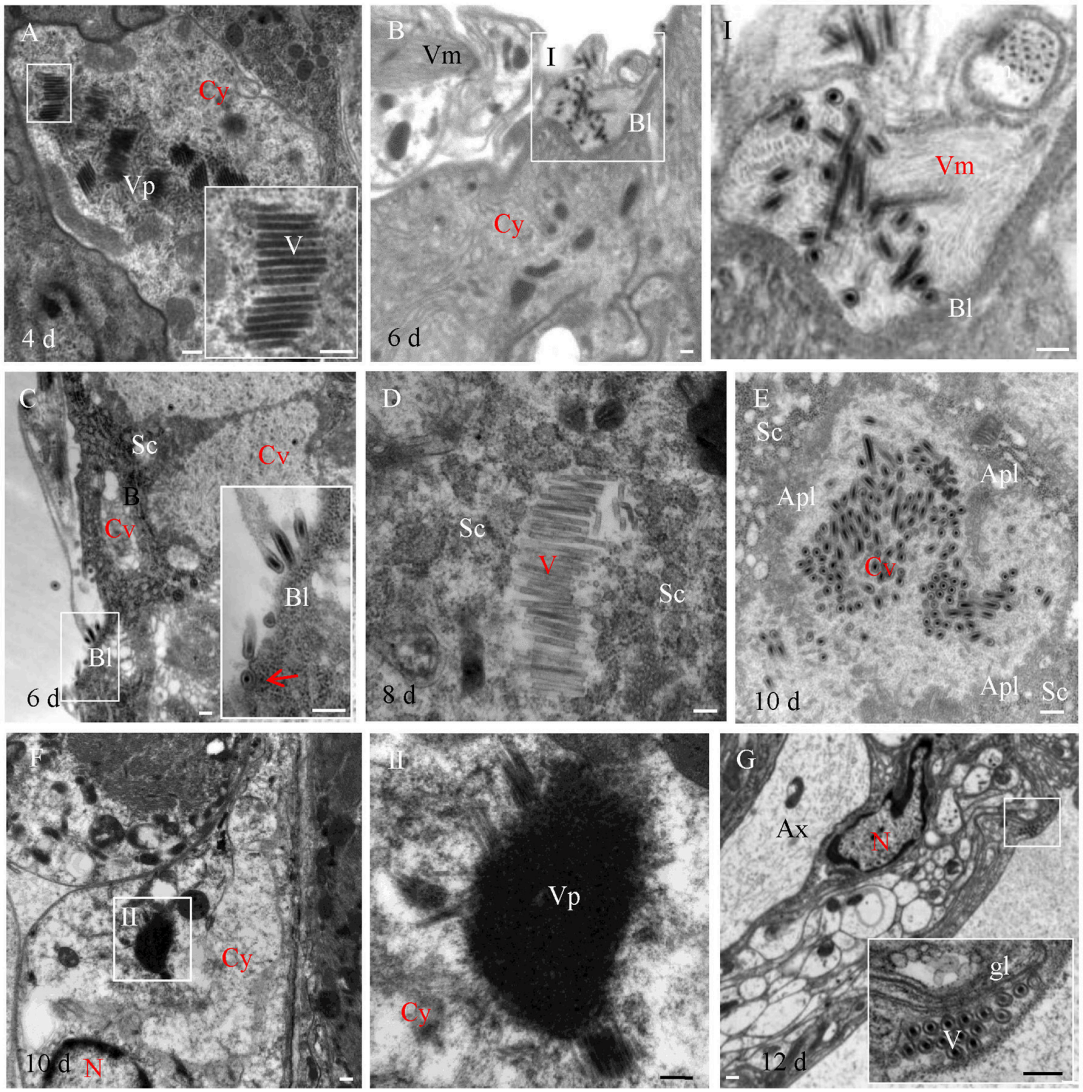
Electron microscopy showing infection of RSMV in leafhopper *R. dorsalis*. **(A)** RSMV formed viroplasm and non-enveloped viral particles in the cytoplasm of the filter chamber epithelium. **(B)** Enveloped virus particles distributed in the visceral muscle. Panel I was the enlarged image of the boxed area in panel **(B)**. **(C)** Enveloped virus particles infected the salivary glands. Arrow indicates the endocytosis of RSMV. **(D)** RSMV replicated in the cytoplasm of the salivary glands. **(E)** RSMV particles assembled in the cavity of salivary gland. **(F)** RSMV infected and formed viroplasm in the glial cells of the central nervous system. Panel II was the enlarged image of the boxed area in **(F)**. **(G)** Enveloped virus particles distributed in the glial layer of the central nervous system. Apl, apical plasmalemma; Ax, axon; Bl, basal lamina; Cy, cytoplasm; Cv, cavity; Fc, filter chamber; Gl, glial layer; Sc, salivary cytoplasm; Sg, salivary glands; V, virus particle; Vm, visceral muscle; Vp, viroplasm. Bars, 100 nm.

To further reveal the infection characteristics of RSMV in *R. dorsalis*, the localization of RSMV in the intestine, salivary gland and central nerve of *R. dorsalis* was observed with electron microscopy. We found that RSMV formed viroplasm in the cytoplasm of midgut epithelium and a large number of non-enveloped virus particles arranged around the viroplasm (Figure 3A). Then RSMV particles passed through the basal lamina from midgut epithelium toward visceral muscle tissues, where they existed as enveloped virus particles (Figure 3B). When RSMV spread to the surface of salivary glands, the enveloped virus particles invaded into the salivary gland cells from the basal lamina by the endocytosis-like mechanism (Figure 3C), and formed viroplasm and non-enveloped virus particles in the salivary cytoplasm (Figure 3D). Finally, the virus particles were released into the gland cavity and became enveloped (Figure 3E). In the central nervous system, RSMV particles were infected the glial cytoplasm and formed viroplasm and non-enveloped virus particles (Figure 3F). However, Then enveloped virus particles were distributed in the glial layer (Figure 3G), but none were found in the axons of the central nervous system. These results demonstrated that RSMV particles displayed as non-enveloped form when propagating in cytoplasm of different tissues, and became enveloped when spread within insect body. Meanwhile, RSMV could spread directly into the central nervous system via glial cells, but not spread within the axons.

### The Leafhopper *Nephotettix virescens* Is a New Vector Insect for RSMV

**Figure 4:**
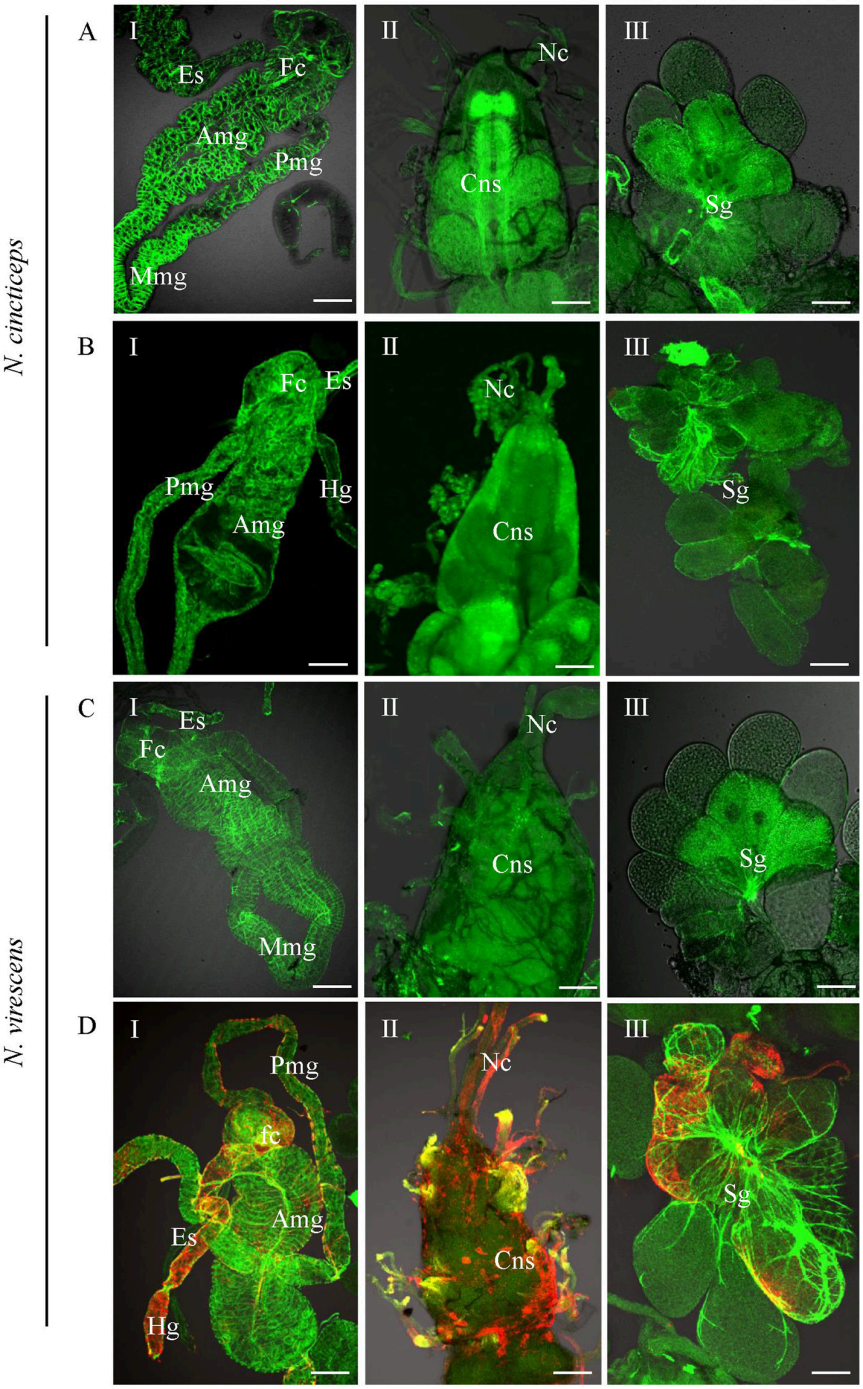
Infection of RSMV in the leafhoppers *Nephotettix cincticeps* and *Nephotettix virescens* at 20 days padp. **(A,C)** Leafhoppers fed on healthy plants. **(B,D)** Leafhoppers fed on RSMV-infected plants. The internal organs (panel I), central nervous system (panel II) and salivary glands (panel III) of leafhoppers were stained for viral antigen with N–rhodamine (red) and for actin with phalloidin–Alexa Fluor 488 carboxylic acid (green) and examined by confocal microscopy. Amg, anterior midgut; Cns, central nervous system; Es, esophagus; Fc, filter chamber; Hg, hindgut; Mmg, middle midgut; Mv, microvilli; Nc, nerve cord; Pmg, posterior midgut; Sg, salivary glands. Bars, 100 nm.

To determine the possibility of acquisition and transmission of RSMV by the leafhopper *Nephotettix virescens* and *Nephotettix cincticeps*, we allowed second-instar nymphs from the non-viruliferous population of leafhoppers to feed for 2 days on RSMV-infected rice plants. Insects then were reared for 14 days on healthy rice seedlings and analyzed by immunofluorescence staining with N-FITC and RT-PCR as previously. At 14-day padp, N protein was not observed in the body of *N. cincticeps* that fed on healthy or RSMV-infected plants (Figures 4A,B, Table 2). At this time, No signal for the RSMV antigen was observed in body of *N. virescens* that fed on healthy plants (Figure 4C). However, N proteins were densely visualized in the digestive systems, compound ganglionic mass, and salivary glands in ca. Ten percent of the tested leafhopper *N. virescens* (Figure 4D, Table 2). Meanwhile, the viruliferous *N. virescens* all had the ability to transmit RSMV to rice plants (Table 2). Thus, these results indicated that the leafhopper *N. virescens* is a new vector for RSMV.

**Table 2:** The RSMV acquisition and transmission effeciency of *Nephotettix virescens* and *Nephotettix cincticeps* when feeding on RSMV-infected rice plants.

**Leafhopper**	**Methods**	**RSMV acquisition rate**		**RSMV transmission rate**	
		**No. of viruliferous insect**	**Acquisition rate (%)**	**No. of transmission insect**	**Transmission rate (%)**
*Nephotettix virescens*	Immunofluorescence	5/46	10.9		
		5/48	10.4		
		4/42	9.5		
	RT-PCR	4/50	8.0	5/46	10.9
		4/47	8.5	5/48	10.4
		5/48	10.4	4/42	9.5
*Nephotettix cincticeps*	Immunofluorescence	0/48	0		
		0/45	0		
		0/50	0		
	RT-PCR	0/46	0	0/48	0
		0/50	0	0/45	0
		0/45	0	0/50	0

## Discussion

In general, the replication of rhabdoviruses is thought to be initiated after the N protein accumulates to a sufficient concentration to mediate a switch from transcription to replication (Dietzgen, 2012). RSMV as a new cytorhabdovirus replicated and virion accumulated in the cytoplasm of infected rice (Yang et al., 2017a). In the present study, by using VCMs derived from *R. dorsalis*, our cytopathological analysis revealed that protein N of RSMV localized in the cytoplasm of infected VCMs and formed punctate inclusions. Meanwhile, the N antibodies specifically reacted with punctate inclusions and virus particles (Figure 1G). These results provide direct evidence that RSMV replicates in cytoplasm and the N protein is not only a component of virus particle, but also a component of the viroplasm matrix.

RSMV as an insect-borne propagative plant virus has the potential to seriously threaten the stability of rice production in China (Yang et al., 2017a). Thus, characterization of the viral infection and transmission mechanisms in insect are significant for designing strategies to interfere with viral transmission. Recently, the transmission biology of rice stripe mosaic virus by the leafhopper *R. dorsalis* has been investigated (Yang et al., 2017b). However, the infection mechanism of RSMV in the leafhopper vector is still unknown. Our work provides the direct evidence for the infection route of RSMV in various organs and tissues of its insect vector *R. dorsalis* using immunofluorescence microscopy and electron microscopy. Our results showed that epithelial cells of the filter chamber were susceptible to RSMV infection as early as 2 days padp, suggesting that the epithelial cells of the filter chamber were the initial infection sites for RSMV entry and accumulation (Figure 2B). This finding revealed a similar initial infection site for other phytoreoviruses, such as rice dwarf virus (RDV) and rice gall dwarf virus, in the body of their leafhopper vectors (Chen et al., 2011; Zheng et al., 2015). However, the initial infection site for other member of rhabdovirus, such as barley yellow striate mosaic virus and maize mosaic virus were hidgut and midgut of their vector planthoppers, respectively (Ammar and Hogenhout, 2008; Cao et al., 2018). These difference indicated that every persistent-propagative plant virus has a tissue tropism of virus entry among vector insect. Usually, after replicated in initially infected cells, persistent-propagative viruses need to be transported across the epithelial cells in gut to salivary glands (Hogenhout et al., 2008). Previous studies have demonstrated that viruses used different routes, such as hemocoel, neurotropic, tracheae, visceral muscles, or ligament-like tissue, to directly or indirectly spread into the salivary glands (Romoser et al., 2004; Ammar and Hogenhout, 2008; Jia et al., 2014; Montero-Astúa et al., 2016). With regard to RSMV, infection of the suspensory ligament of *R. dorsalis* was much more extensive and occurred much earlier than that of other tissues. In addition, as early as 6 days padp, RSMV spread from the epithelial cells to the visceral muscle tissues of the filter chamber (Figure 2C), from where they moved along the suspensory ligament to the salivary glands (Figures 2D,E, Table 1). We speculate that RSMV may have evolved to avoid transmission barriers in *R. dorsalis* by proceeding to salivary glands via the suspensory ligament. These results also indicated that the minimum latent period of RSMV in *R. dorsalis* was 6 days which was consistent with the previous report (Yang et al., 2017b). Moreover, RSMV only infected the surface cells of the reproductive system of *R. dorsalis*, but did not infect into cytoplasm of the oocyte or cavity of the testis and seminal vesicle (Supplementary Figure 1) and did not transmit to the offspring leafhoppers (Supplementary Table 1). These results indicating that the failure of *R. dorsalis* to vertically transmit RSMV was due to the strong membrane or tissues barriers in the reproductive system.

As a plant infecting rhabdovirus, RSMV also could infect the compound ganglionic mass and nerve cord of the central nervous system (Figure 2I). The infection of RMSV in the nerve cord suggested that RSMV may proceed from the central nervous system to the closely associated tissues via the nerve cord. This neurotropic characteristic is similar with that of some rhabdoviruses, such as rabies virus (an animal rhabdovirus infecting primates) and maize mosaic virus (a planthopper-transmitted nucleorhabdovirus; Ammar and Hogenhout, 2008; Ugolini, 2011). However, unlike rabies virus dissemination in the axon (Ugolini, 2011), the enveloped RSMV particles only distributed and spread in the “myelin sheath” of the central nervous system (Figure 3G). Furthermore, unlike maize mosaic virus spread from the anterior diverticulum and esophagus to the salivary glands via the nervous system (Ammar and Hogenhout, 2008), the infection of RSMV in the nervous system was later than in other tissues such as the suspensory ligament and salivary glands. Collectively, these findings provide strong evidence that RSMV uses a different mechanism to spread in the central nervous system, which substantially extends our understanding of the transmission mechanism of rhabdoviruses.

The vector specificity of plant viruses is common in nature, many plant viruses are transmitted by a specific or a few closely related insect species (Hogenhout et al., 2008; Ammar et al., 2009). The specificity of a plant virus for its vector can be explained by transmission barriers posed by different tissues in insects (Hogenhout et al., 2008). It has been demonstrated previously that RSMV is transmitted efficiently by *R. dorsalis*, and the closely related species *N. cincticeps* also can carry RSMV, but can't transmit it (Yang et al., 2017b). In this study, we found that the leafhopper *N. virescens* collected from the field exhibited a low RSMV-positive rate (~10%). And RSMV not only infected the alimentary canal, but also the salivary glands of *N. virescens* (Figure 4). Particularly, transmission assays showed that the viruliferous *N. virescens* was able to transmit RSMV to healthy plants (Table 2). We thus believe that *N. virescens* is a new vector for RSMV. However, *N. cincticeps*, collected from Xinning, Guangdong and bred in laboratory for about 8 years, could not carry RSMV when fed on RSMV-infected rice plants for only 2 d. According to the field survey of Yang et al. (2017b) and our results, we deduced that *N. cincticeps* might need a longer viral acquisition access period to attain a lower acquisition rate, but could not transmit RSMV to healthy plants (Yang et al., 2017b). Whether *N. cincticeps* is an additional vector of RSMV need further investigation. In fact, leafhopper *N. virescens* can transmit RDV (family Reoviridae), rice yellow stunt virus (family Rhabdoviridae), and rice tungro-associated viruses (Hsieh et al., 1970; Xie et al., 1981; Hibino, 1983). It is also one of the dominating pests in winter rice field in Guangdong, China (Zhong et al., 2004). Therefore, we deduced that *N. virescens* might be one of the overwintering hosts and evolve to be an efficient vector for RSMV during the continued evolution. Thus, close monitoring of the capability of *N. virescens* to transmit RSMV in field is necessary.

## Author Contributions

DJ and TW conceived and designed the experiments. PZ, XS, PL, JS, and YY performed the experiments. PZ maintained insect cell line. DJ, PZ, and XS analyzed the data. DJ and JW wrote the manuscript. All authors read and approved the final manuscript.

### Conflict of Interest Statement

The authors declare that the research was conducted in the absence of any commercial or financial relationships that could be construed as a potential conflict of interest.

## Supplementary Material

Supplementary Table 1 The RSMV-positive R. dorsalis nymphs propagated from RSMV positive females.

Supplementary Figure 1 Infection of RSMV in the female **(A)** and male **(B)** reproductive systems of leafhopper *R. dorsalis*. The reproductive systems of leafhopper *R. dorsalis* were stained for viral antigen with N–rhodamine (red) and for actin with phalloidin–Alexa Fluor 488 carboxylic acid (green) and examined by confocal microscopy. Panels I, II, and III were the enlarged images of the boxed areas in panels **(A,B)**, respectively. Ag, accessory gland; Fc, follicular cell; Le, lateral ejaculatory duct; O, oocyte; Pd, pedicel; Sv, seminal vesicle; T, testes; Vd, vas deferens. Bars, 100 nm.

Supplementary Figure 2 Infection of RSMV in the hemocytes of leafhopper *R. dorsalis*. The hemocytes of healthy **(A)** and viruliferous leafhopper *R. dorsalis* at 4**(B)**, 6**(C)**, 8**(D)**,12**(E)** days padp were stained for viral antigen with N–FITC (green) and DAPI (blue) and examined by confocal microscopy. Bars, 5μm.

Supplementary Figure 3 Infection route of RSMV in the insect vector. The tissues of leafhopper *R. dorsalis* were displayed as the bright-field images with N-rhodamine.

## References

[B1] Ammar E-D. Hogenhout S. A. (2008). A neurotropic route for Maize mosaic virus (*Rhabdoviridae*) in its planthopper vector Peregrinus maidis. Virus Res. 131, 77–85. 10.1016/j.virusres.2007.08.010 17928084

[B2] Ammar E-D. Tsai C. W. Whitfield A. E. Redinbaugh M. G. Hogenhout S. A. (2009). Cellular and molecular aspects of rhabdovirus interactions with insect and plant hosts. Annu. Rev. Entomol. 54, 447–468. 10.1146/annurev.ento.54.110807.090454 18793103

[B3] Cao Q. Xu W. Y. Gao Q. Jiang Z. H. Liu S. Y. Fang X. D.. (2018). Transmission characteristics of barley yellow striate mosaic virus in its planthopper vector *Laodelphax striatellus*. Front. Microbiol. 9:1419. 10.3389/fmicb.2018.01419 30008708 PMC6034074

[B4] Chen H. Chen Q. Omura T. Uehara-Ichiki T. Wei T. (2011). Sequential infection of Rice dwarf virus in the internal organs of its insect vector after ingestion of virus. Virus Res. 160, 389–394. 10.1016/j.virusres.2011.04.028 21570430

[B5] Chen H. Zheng L. Jia D. Zhang P. Chen Q. Liu Q. (2013). Rice gall dwarf virus exploits tubules to facilitate viral spread among cultured insect vector cells derived from leafhopper *Recilia dorsalis*. Front. Microbial. 23:206 10.3389/fmicb.2013.00206 PMC3719018 23888157

[B6] Chen Q. Chen H. Mao Q. Liu Q. Shimizu T. Uehara-Ichiki T.. (2012). Tubular structure induced by a plant virus facilitates viral spread in its vector insect. PLoS Pathog. 8:e1003032. 10.1371/journal.ppat.1003032 23166500 PMC3499585

[B7] Chen Q. Wei T. (2016). Viral receptors of the gut: insect-borne propagative plant viruses of agricultural importance. Curr. Opin. Insect Sci. 16, 9–13. 10.1016/j.cois.2016.04.014 27720057

[B8] Chen Y. Lu C. Li M. Wu W. Zhou G. Wei T. (2016). Adverse effects of Rice gall dwarf virus upon its insect vector *Recilia dorsalis* (Hemiptera: Cicadellidae). Plant Dis. 100, 1–7. 10.1094/PDIS-06-15-0713-RE 30688603

[B9] Dietzgen R. G. (2012). Morphology, genome organization, transcription and replication of rhabdoviruses, in Rhabdoviruses: Molecular Taxonomy, Evolution, Genomics, Ecology, Host–Vector Interactions, Cytopathology and Control, eds Dietzgen R. G. Kuzmin I. V. (Norfolk, FL: Caister Academic Press), 5–12.

[B10] Hibino H. (1983). Transmission of two rice tungro-associated viruses and rice waika virus from doubly or singly infected source plants by leafhopper vectors. Plant Dis. 67, 774–777. 10.1094/PD-67-774

[B11] Hogenhout S. A. Ammarel D. Whitfield A. E. Redinbaugh M. G. (2008). Insect vector interactions with persistently transmitted viruses. Annu. Rev. Phytopathol. 46, 327–359. 10.1146/annurev.phyto.022508.092135 18680428

[B12] Hsieh S. P. Y. Chiu R. J. Chen C. C. (1970). Transmission of rice transitory yellowing virus by *Nephotettix impicticeps*. Phytopathol. 60:1534.

[B13] Jia D. Chen H. Mao Q. Liu Q. Wei T. (2012a). Restriction of viral dissemination from the midgut determines incompetence of small brown planthopper as a vector of Southern rice black-streaked dwarf virus. Virus Res. 167, 404–408. 10.1016/j.virusres.2012.05.023 22683297

[B14] Jia D. Chen Q. Mao Q. Zhang X. Wu W. Chen H.. (2018). Vector mediated transmission of persistently transmitted plant viruses. Curr. Opin. Virol. 28, 127–132. 10.1016/j.coviro.2017.12.004 29306179

[B15] Jia D. Guo N. Chen H. Akita F. Xie L. Omura T.. (2012b). Assembly of the viroplasm by viral non-structural protein Pns10 is essential for persistent infection of rice ragged stunt virus in its insect vector. J. Gen. Virol. 93, 2299–2309. 10.1099/vir.0.042424-0 22837415

[B16] Jia D. Mao Q. Chen H. Wang A. Liu Y. Wang H.. (2014). Virus-induced tubule: a vehicle for rapid spread of virions through basal lamina from midgut epithelium in the insect vector. J. Virol. 88, 10488–10500. 10.1038/nmicrobiol.2017.25 24965461 PMC4178856

[B17] Liao Z. Mao Q. Li J. Lu C. Wu W. Chen H.. (2017). Virus-induced tubules: a vehicle for spread of virions into ovary oocyte cells of an insect vector. Front. Microbiol. 8:475. 10.3389/fmicb.2017.00475 28382031 PMC5360704

[B18] Luo M. Green T. J. Zhang X. Tsao J. Qiu S. (2007). Structural comparisons of the nucleoprotein from three negative strand RNA virus families. Virol. J. 4:72. 10.1186/1743-422X-4-72 17623082 PMC2031895

[B19] Mao Q. Liao Z. Li J. Liu Y. Wu W. Chen H.. (2017). Filamentous structures induced by a phytoreovirus mediate viral release from salivary glands in its insect vector. J. Virol. 91:e00265–17. 10.1128/JVI.00265-17 28381575 PMC5446657

[B20] Montero-Astúa M. Ullman D. E. Whitfield A. E. (2016). Salivary gland morphology, tissue tropism and the progression of tospovirus infection in *Frankliniella occidentalis*. Virology 493, 39–51. 10.1016/j.virol.2016.03.003 26999025

[B21] Romoser W. S. Wasieloski L. P. Jr. Pushko P. Kondig J. P. Lerdthusnee K. Neira M.. (2004). Evidence for arbovirus dissemination conduits from the mosquito (Diptera: Culicidae) midgut. J. Med. Entomol. 41, 467–475. 10.1603/0022-2585-41.3.467 15185952

[B22] Ugolini G. (2011). Rabies virus as a transneuronal tracer of neuronal connections. Adv. Virus Res. 79:165–202. 10.1016/B978-0-12-387040-7.00010-X 21601048

[B23] Walker P. J. Dietzgen R. G. Joubert D. A. Blasdell K. R. (2011). Rhabdovirus accessory genes. Virus Res. 162, 110–125. 10.1016/j.virusres.2011.09.004 21933691 PMC7114375

[B24] Wei T. Uehara-Ichiki T. Miyazaki N. Hibino H. Iwasaki K. Omura T. (2009). Association of Rice gall dwarf virus with microtubules is necessary for viral release from cultured insect vector cells. J. Virol. 83:10830–10835. 10.1128/JVI.01067-09 19640979 PMC2753141

[B25] Whitfield A. E. Ullman D. E. German T. L. (2005). Tospovirus-thrips interactions. Annu. Rev. Phytopathol. 43, 459–489. 10.1146/annurev.phyto.43.040204.140017 16078892

[B26] Wu W. Zheng L. Chen H. Jia D. Li F. Wei T. (2014). Nonstructural protein NS4 of Rice stripe virus plays a critical role in viral spread in the body of vector insects. PLoS ONE 9:e88636. 10.1371/journal.pone.0088636 24523924 PMC3921211

[B27] Xie L. H. Lin J. Y. Guo J. R. (1981). A new insect vector of rice dwarf virus. Int. Rice Res. Newsl. 6:14.

[B28] Yang X. Huang J. Liu C. Chen B. Zhang T. Zhou G. (2017a). Rice stripe mosaic virus, a novel Cytorhabdovirus infecting rice via leafhopper transmission. Front. Microbiol. 7:2140. 10.3389/fmicb.2016.02140 28101087 PMC5210121

[B29] Yang X. Zhou T. Chen B. Zhou G. (2017b). Transmission biology of Rice stripe mosaic virus by an efficient insect vector *Recilia dorsalis* (Hemiptera: Cicadellidae). Front. Microbiol. 8:2457. 10.3389/fmicb.2017.02457 29312171 PMC5732235

[B30] Zheng L. Chen H. Liu H. Xie L. Wei T. (2015). Assembly of viroplasms by viral nonstructural protein Pns9 is essential for persistent infection of rice gall dwarf virus in its insect vector. Virus Res. 196, 162–169. 10.1016/j.virusres.2014.11.025 25455335

[B31] Zhong Y. Luo Z. Luo S. Lan L. (2004). Occurrence regularity and control of rice gall dwarf disease. China Plant Protect. 24, 14–15.

