# Supplementary material for: Infection Characteristics of Rice Stripe Mosaic Virus in the Body of the Vector Leafhoppers
Source: Front Microbiol. 2019 Jan 8;9:3258. doi: 10.3389/fmicb.2018.03258 (PMC6331539; doi:10.3389/fmicb.2018.03258)
Supplement: Supplementary Table 1 — The RSMV-positive R. dorsalis nymphs propagated from RSMV positive females. [file Table_1.DOC]

Supplementary Table 1. The RSMV-positive *R. dorsalis* nymphs propagated from RSMV positive females.

| Methods | RSMV positive rate | | |
| --- | --- | --- | --- |
|  | No. of tested | No. of viruliferous insect | Positive rate (%) |
| Immunofluorescence | 100 | 0 | 0 |
|  | 95 | 0 | 0 |
|  | 93 | 0 | 0 |
| RT-PCR | 80 | 0 | 0 |
|  | 92 | 0 | 0 |
|  | 86 | 0 | 0 |
