# Supplementary figures and images for: Infection Characteristics of Rice Stripe Mosaic Virus in the Body of the Vector Leafhoppers
Source: Front Microbiol. 2019 Jan 8;9:3258. doi: 10.3389/fmicb.2018.03258 (PMC6331539; doi:10.3389/fmicb.2018.03258)

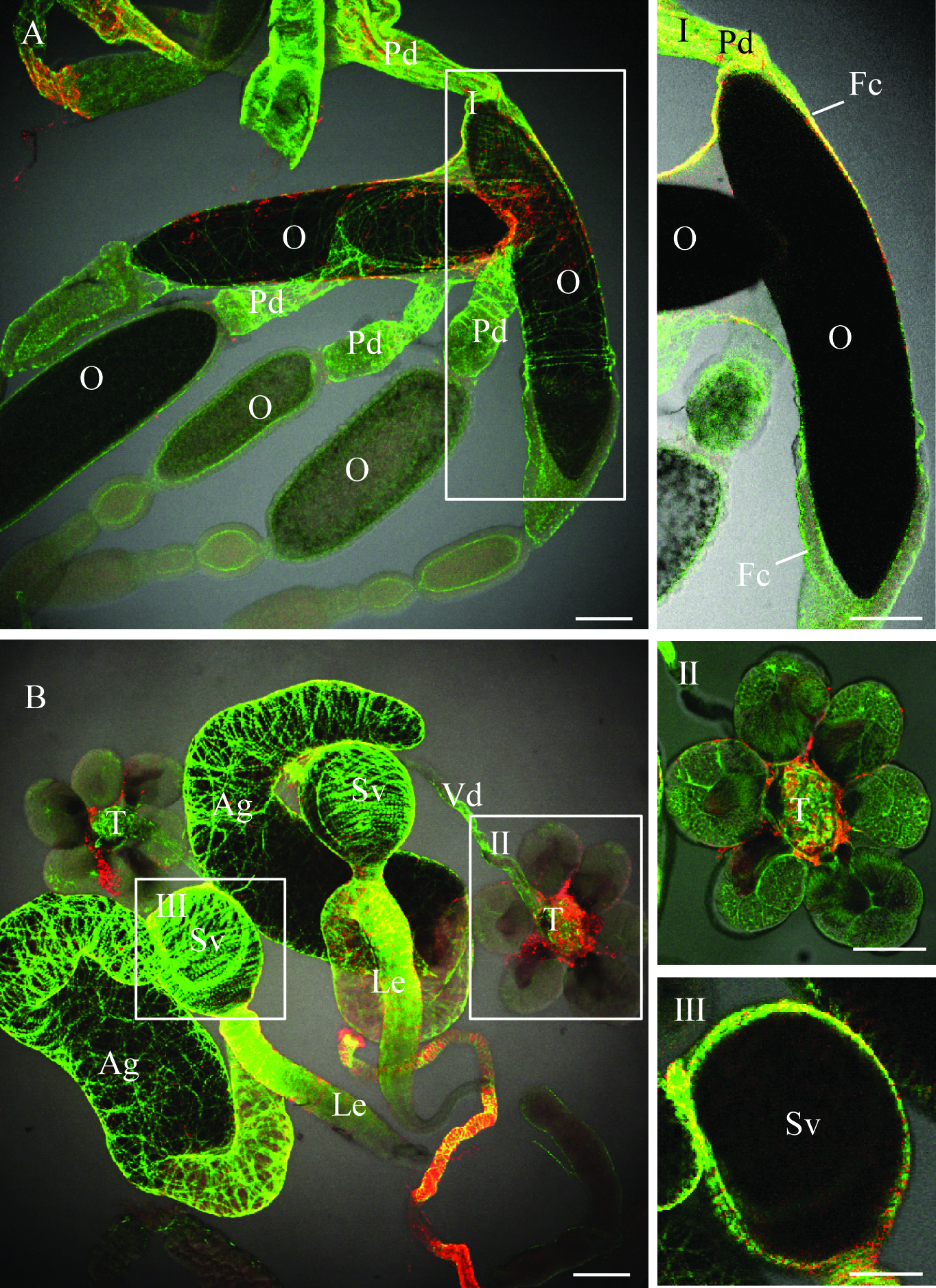

Supplement: Supplementary Figure 1 — Infection of RSMV in the female (A) and male (B) reproductive systems of leafhopper R. dorsalis. The reproductive systems of leafhopper R. dorsalis were stained for viral antigen with N–rhodamine (red) and for actin with phalloidin–Alexa Fluor 488 carboxylic acid (green) and examined by confocal microscopy. Panels I, II, and III were the enlarged images of the boxed areas in panels (A,B), respectively. Ag, accessory gland; Fc, follicular cell; Le, lateral ejaculatory duct; O, oocyte; Pd, pedicel; Sv, seminal vesicle; T, testes; Vd, vas deferens. Bars, 100 nm. [file Image_1.JPEG]

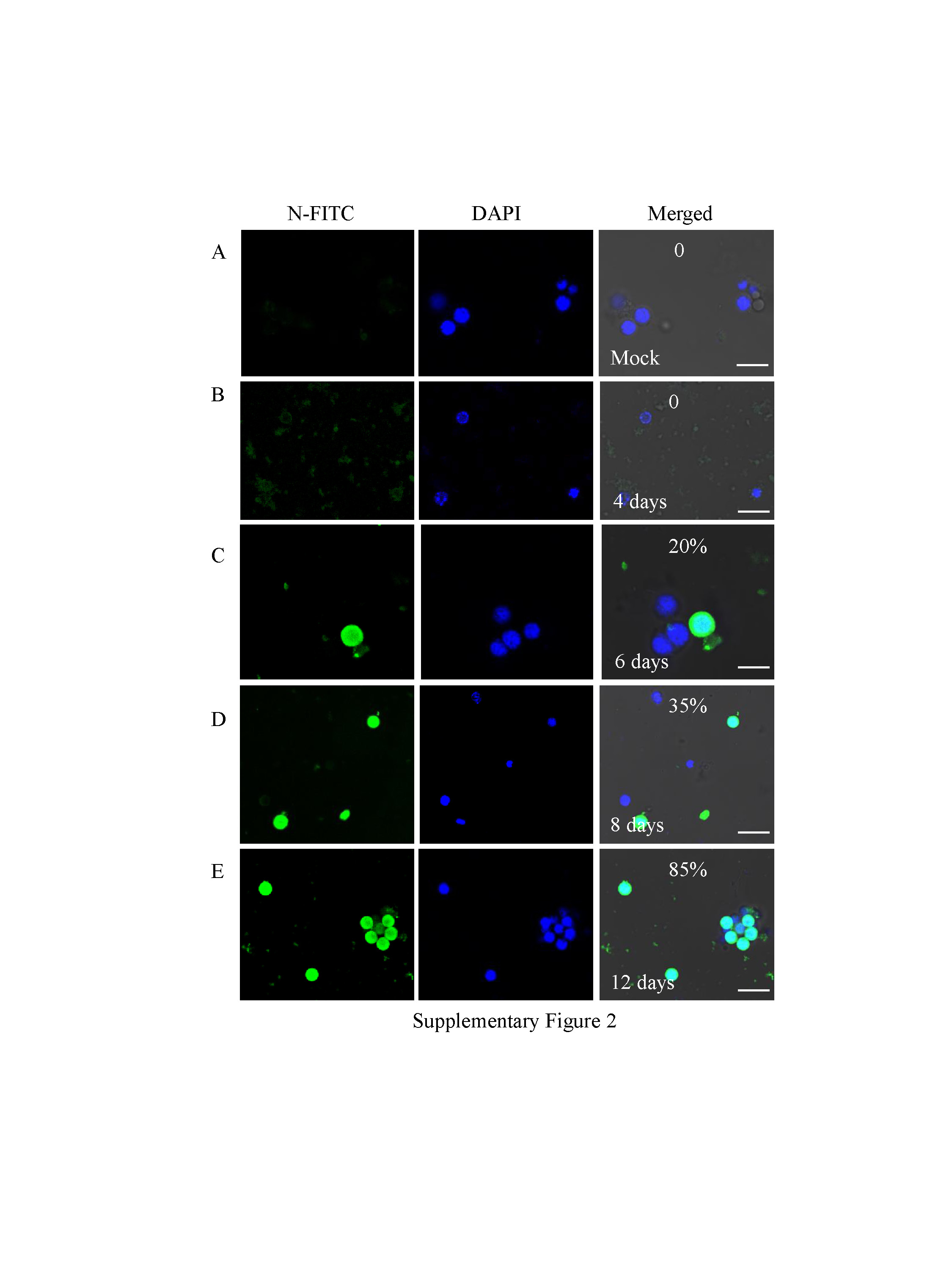

Supplement: Supplementary Figure 2 — Infection of RSMV in the hemocytes of leafhopper R. dorsalis. The hemocytes of healthy (A) and viruliferous leafhopper R. dorsalis at 4(B), 6(C), 8(D),12(E) days padp were stained for viral antigen with N–FITC (green) and DAPI (blue) and examined by confocal microscopy. Bars, 5μm. [file Image_2.JPEG]

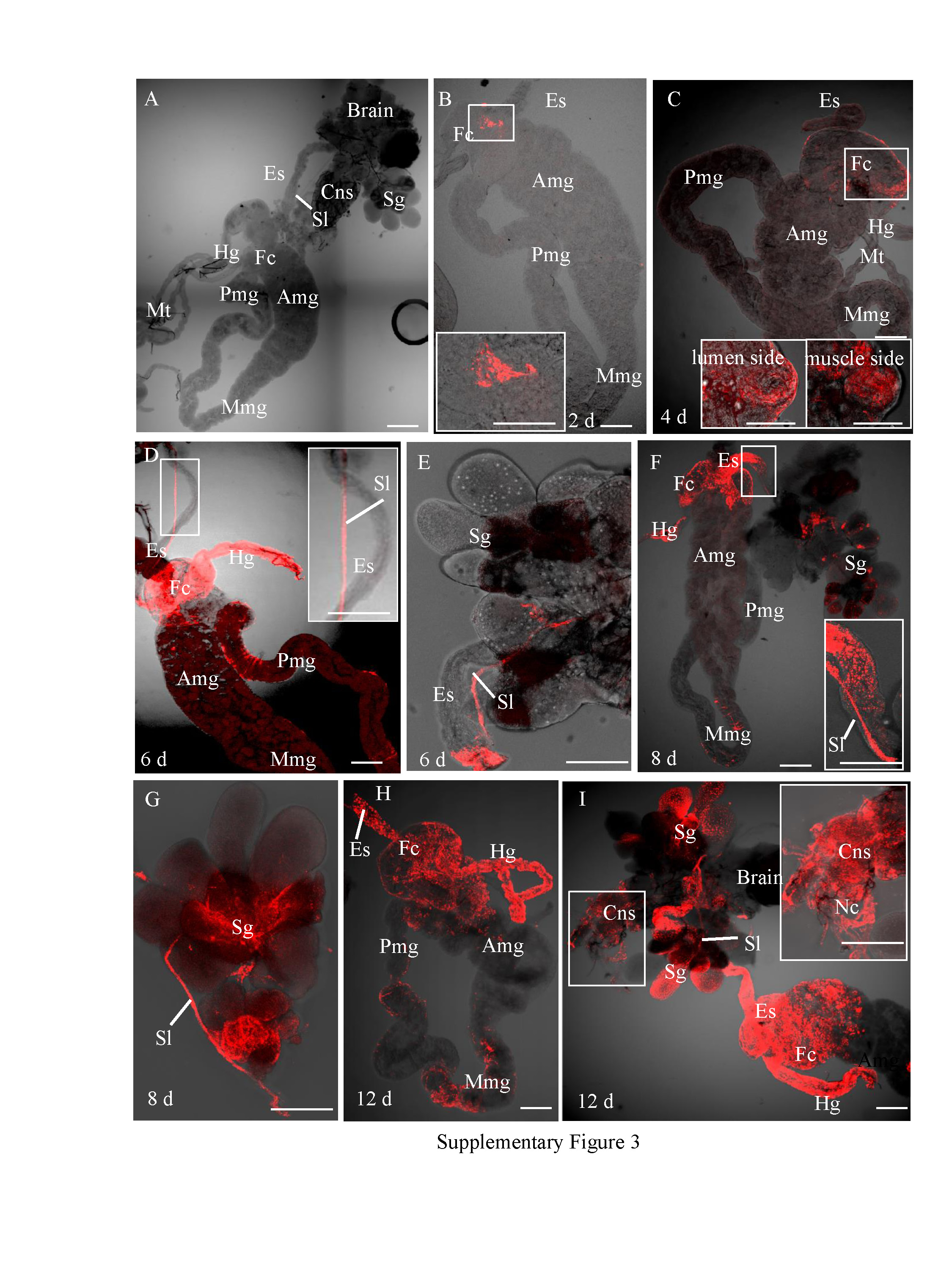

Supplement: Supplementary Figure 3 — Infection route of RSMV in the insect vector. The tissues of leafhopper R. dorsalis were displayed as the bright-field images with N-rhodamine. [file Image_3.JPEG]
